# Strength of motivation of 1^st^ year dental students of a dental institution: A cross-sectional study

**DOI:** 10.12669/pjms.40.7.9112

**Published:** 2024-08

**Authors:** Fizzah Ali, Muhammad Tauqeer Ehsan, Fatima Suhaib, Mariam Fatima

**Affiliations:** 1 Fizzah Ali Demonstrator, Department of Dental Education, Lahore Medical & Dental College, Lahore, Pakistan; 2 Muhammad Tauqeer Ehsan Assistant Professor, Department of Dental Education, Lahore Medical & Dental College, Lahore, Pakistan; 3 Fatima Suhaib Assistant Professor, Department of Dental Materials, Lahore Medical & Dental College, Lahore, Pakistan; 4 Mariam Fatima Assistant Professor, Department of Community & Preventive Dentistry, Lahore Medical & Dental College, Lahore, Pakistan

**Keywords:** Motivation, Dental students, Strength of Motivation for Medical School (SMMS) questionnaire

## Abstract

**Objective:**

The study aims to evaluate the motivation levels of dental students, with an emphasis on first-year BDS students, by utilizing the Strength of Motivation for Medical School (SMMS) questionnaire.

**Methods:**

It was a descriptive cross-sectional quantitative study targeting 89 first-year BDS students enrolled at Lahore Medical and Dental College from 5^th^ June to 18^th^ August 2023. The Strength of Motivation for Medical School (SMMS) questionnaire was given to the participants after ethical board approval. Data analysis was done through SPSS version 26. The SMMS score was presented as the means standard deviation and an independent t-test was used to find the difference between the groups. The maximum score possible is 80 and the minimum is 16. The higher the score, the greater the strength of motivation.

**Results:**

In this study, a total of 89 first-year BDS students completed motivation questionnaires, with 34.8% males and 65.2% females. The average age was 19.92 ± 3.13. The overall Strength of Motivation Score (SMMS) averaged 45.53 ± 6.82. Results indicated 9% low, 89.9% moderate, and 1.1% strong motivation. Females had a slightly higher mean SMMS (45.93 ± 6.88) than males (44.80 ± 6.76), but the difference was deemed insignificant (p = 0.462) via independent t-test.

**Conclusion:**

Motivation is vital to achieving excellence in academic pursuits. Nevertheless, there isn’t a single criterion that can be utilized to assess success and motivation. Our primary focus must be on every possible outcome of success, not just the scoring criteria.

## INTRODUCTION

Dentistry is a constantly evolving field in which students must possess drive and dedication. Success in any discipline, including dentistry, is largely dependent on motivation. People who possess driving power can persevere through difficult situations to accomplish their goals. Academic success, professional growth, and patient care are all greatly influenced by motivation.^1^ To effectively support dentistry students and better understand their educational needs, it is imperative to evaluate the level of motivation among them.

Like any other field, the success of dental students is largely dependent on their motivation levels. The rigorous nature of dentistry necessitates devotion and diligence; therefore, motivation is especially important. Thus, assessing dental students’ motivation levels is essential to comprehending the elements affecting their success in this discipline.^2^ Personality qualities and features of students are directly associated with different types of motivation, which helps them succeed academically throughout their schooling. Nonetheless, there isn’t a lot of investigation conducted on the unique motivational factors that this particular university offers dental.^3^

Many studies have been done to highlight the importance of motivation in dental education. Orsini et al. found that motivation significantly predicted academic performance among dental students.^4^ Similarly, Kusurkar et al. reported that motivation was positively associated with academic achievement and satisfaction with the dental curriculum.^5^ In addition, other studies have demonstrated that motivation is essential for maintaining high levels of professional engagement and improving patient care outcomes.^6^

Various tools and instruments have been developed to measure the strength of motivation among dental students. One such instrument is the Strength of Motivation for Medical School (SMMS) questionnaire developed by Kusurkar et al. and Tencate et al.^5^ This questionnaire assesses the strength of motivation for medical school and has been validated in several studies.^7^-^10^ It consists of 30 items divided into three subscales: intrinsic motivation, extrinsic motivation, and social motivation. Although the SMMS questionnaire has been used in various studies to assess motivation among medical students, its use in dental education has been limited. Several studies have demonstrated that academic achievement in dental education and motivation are positively correlated.^8^,^11^

Knowledge of how dental students’ motivation and academic performance relate to one another can help to pinpoint possible areas for development and aid help them reach their objectives. Since the first year of dentistry school sets the foundation for the rest of the educational program, it is particularly significant. To excel in this discipline, students must have a strong sense of motivation because the coursework is tough, time-consuming, and challenging. Therefore, it is important to assess the strength of motivation of first-year dental students to identify potential areas of improvement and to provide support to help them achieve their goals.

Therefore, this study aimed to assess the strength of motivation for 1st-year BDS (Bachelor of Dental Surgery) students using the SMMS questionnaire. The objective of our study was to find the level of motivation of 1^st^ Year BDS students at Lahore Medical and Dental College (LMDC) and find the difference in motivation levels between males and females. In order to create effective methods that boost dental students’ motivation and success in the profession, it is crucial to comprehend the relative contributions of intrinsic and extrinsic motivation to their performance.

## METHODS

This cross-sectional descriptive study was done in Lahore Medical and Dental College from 5^th^ June to 18^th^ August 2023. There were 89 students based on convenience sampling. Informed consent was obtained from all participants outlining the purpose of the study, data confidentiality and voluntary participation. The Strength of Motivation for Medical School (SMMS) questionnaire was employed. The SMMS is a validated tool designed to assess motivation levels and their determinants among medical and dental students. Non-probability sampling was done as all students of 1^st^ Year BDS participated in the study.

### Ethical approval

It was obtained from the Institutional Review Board of the college Ref No. LMDC/FD/2236/23.Date May 16^th^ 2023.

The tool used to determine the strength of motivation was the SMMS questionnaire. The questionnaire was administered through google forms. This questionnaire contains 16 items using the Likert point scale of 1-5 with a range from ‘strongly disagree’ to ‘strongly agree’. The questions have either a positive or negative relation with motivation. Maximum score recorded 80 and minimum was 16. Higher scores showed greater strength of motivation and vice versa. The level of motivation was recorded as low (16-37), moderate (38-58), strong (59-80).^7^

Participants were instructed to respond honestly to the questions, reflecting their motivation for pursuing a BDS degree. The collected data were subjected to descriptive statistical analysis, which involved quantifying and categorizing motivational factors as assessed by the SMMS questionnaire. The SMMS score was presented as means standard deviation and independent t-test was used to find the difference between the groups. Significance was set at 0.05.

## RESULTS

A total of 89 students from first-year BDS participated in the study and filled out the questionnaire Form for Strength of Motivation Scores. There were 31 (34.8%) males and 58 (65.2%) females in the study. The mean age of the participants was 19.92 ± 3.13. The mean value of the SMMS score was 45.53 ± 6.82 for all the participants. The frequency of responses to the questionnaire in males and females and their associated p-value is given in Table-I.

**Table-I:** Frequency of responses given to questions asked in the survey and their associated p-value.

Sr	Questions asked in the survey		Males	Females	P value
1	I would always regret my decision if I hadn’t availed myself of the opportunity to study medicine	Strongly disagree	7	9	0.028
		Disagree	6	15	
		Undecided	9	4	
		Agree	4	19	
		Strongly Agree	5	11	
2	I would quit studying medicine if I were 95% certain that I could never become the specialist of my choice	Strongly disagree	7	8	0.537
		Disagree	10	25	
		Undecided	9	11	
		Agree	4	12	
		Strongly Agree	1	2	
3	I would still choose medicine even if that would mean studying in a foreign country in a language that I have not yet mastered.	Strongly disagree	5	6	0.172
		Disagree	6	12	
		Undecided	10	10	
		Agree	5	23	
		Strongly Agree	5	7	
4	As soon as I would discover that it would take me ten years to qualify as a doctor, I would stop studying	Strongly disagree	6	5	0.081
		Disagree	9	32	
		Undecided	8	8	
		Agree	7	8	
		Strongly Agree	1	5	
5	Even if I could hardly maintain my social life, I would still continue medical training	Strongly disagree	4	2	0.424
		Disagree	5	8	
		Undecided	7	12	
		Agree	12	30	
		Strongly Agree	2	6	
6	I wouldn’t consider any other profession than becoming a doctor	Strongly disagree	7	6	
		Disagree	6	14	
		Undecided	6	11	
		Agree	5	19	
		Strongly Agree	6	8	
7	I would still choose medicine even if that meant I would never be able to go on holidays with my friends anymore	Strongly disagree	9	8	0.301
		Disagree	8	11	
		Undecided	5	13	
		Agree	6	20	
		Strongly Agree	3	5	
8	I would stop studying medicine if I started scoring low marks and failing tests often	Strongly disagree	9	10	0.469
		Disagree	11	32	
		Undecided	5	8	
		Agree	5	6	
		Strongly Agree	1	2	
9	If studying took me more than an average of 60 hours a week, I would seriously consider quitting	Strongly disagree	4	9	0.061
		Disagree	7	27	
		Undecided	11	7	
		Agree	7	12	
		Strongly Agree	2	2	
10	I intend to become a doctor even though that would mean taking CME courses two evenings a week throughout my professional career	Strongly disagree	3	4	0.121
		Disagree	3	5	
		Undecided	13	15	
		Agree	9	31	
		Strongly Agree	3	1	
11	It wouldn’t really bother me too much if I could no longer study medicine	Strongly disagree	2	6	0.391
		Disagree	9	27	
		Undecided	8	8	
		Agree	9	12	
		Strongly Agree	2	4	
12	I would like to become a doctor, even if that would mean giving precedence to my work over my family	Strongly disagree	5	7	
		Disagree	6	13	0.678
		Undecided	10	16	
		Agree	7	18	
		Strongly Agree	2	1	
13	I would quit studying as soon as it becomes apparent that there were no jobs or residents	Strongly disagree	5	6	0.064
		Disagree	4	22	
		Undecided	13	11	
		Agree	7	11	
		Strongly Agree	2	5	
14.	I would not have chosen medicine if it would have caused me to accumulate substantial financial debts	Strongly disagree	5	3	0.071
		Disagree	3	20	
		Undecided	12	17	
		Agree	8	10	
		Strongly Agree	3	5	
15.	I would like to study medicine even if I have to spend a lot of time on topics that later turn out to be a waste of time	Strongly disagree	4	3	0.158
		Disagree	6	12	
		Undecided	10	10	
		Agree	8	28	
		Strongly Agree	3	3	
16.	I would be prepared to retake my final high school exams to get higher marks if this would be necessary to study medicine	Strongly disagree	8	7	0.552
		Disagree	5	12	
		Undecided	9	17	
		Agree	7	18	
		Strongly Agree	2	3	

The mean standard deviation (SD) of the SMMS score in each group and their associated significance is given in Table-II. The strength of motivation scores for all the students showed that 9% had low motivation, 89.9 % had moderate motivation and 1.1% had strong motivation. The level of motivation for all the participants is shown in Fig-I. Females recorded a higher mean SMMS score of 45.93 ± 6.88 as compared to males 44.80 ± 6.76. The Independent t-test showed that the difference between the two groups was insignificant (0.462).

**Table-II:** Mean SMMS score in males and females and difference between the groups.

	Frequency	Mean SMMS Score	Standard Deviation (SD)	Significance
Males	31	44.80	6.76	0.462
Females	58	45.93	6.88	

**Fig.1:**
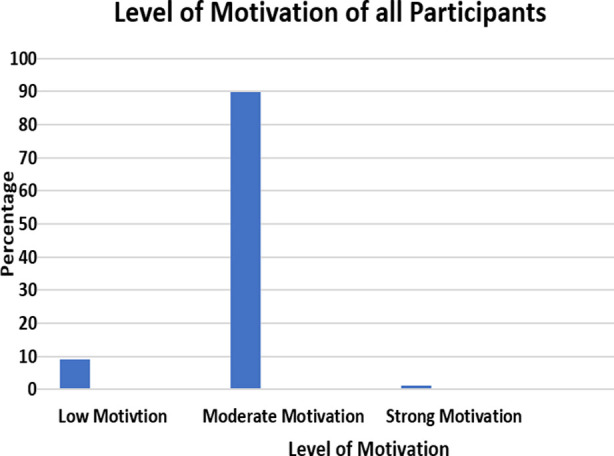
Level of motivation for all the participants.

## DISCUSSION

The mean SMMS score in our study was 45.53 in all the participants. Females had a slightly higher score (45.93) of SMMS as compared to males (44.80) although the difference was not significant (p > 0.05). Literature reported by Kusurkar R et al. and his colleagues’ showed that females were more goal oriented and had higher motivation levels than males who were more focused on career development and securing themselves financially.^13^ However, in a study conducted in China it was found that male students had higher intrinsic motivation than females although females students were performing better academically.^1^ The authors believe that females are more motivated because due to the difference in cultural expectations, females are expected to pursue more stable careers where they will be treated respectfully and dentistry is one such profession.

In order to become a competent and successful medical and dental practitioner, the student needs devotion, motivation and a positive attitude in addition to academic ability.^12^ Factors affecting a student’s motivation levels may depend on personality traits, gender, ethnicity, learning environment, parental support and structure of curriculum.^13^ Studies show that there are a lot of factors that contribute to motivation like earning respect, fame, financial stability and helping people.^13^ It was also observed that internal motivation keeps the spark alive whereas external motivation is short-lived.^1^,^14^

Excellence in dental academia depends on many factors notably being clinical skills, empathy, professionalism, behavior management, communication and critical thinking.^15^ It has been found that there is a positive correlation between internal motivation and better performance.^11^ The results reveal that internal motivation has been a key factor in successful academic record maintenance. The students were asked if they would reconsider their decision to pursue a career in this field and look for other options when they are better aware of the obstacles that they may face. Highly motivated students (1.1%) students from both genders still want to join the dental education as they were willing to sacrifice their family time (31.4%), social life (56.1), and holidays (38.2) to devote time to studying and attending courses (49.4%).

Results show that 89.9% of the students had moderate motivation and their decision to pursue dental education was not affected by long study hours, compromising on leisure time and low scores in exams while 9% of the least motivated participants however see limited specialty options and financial instability as major setbacks. Out of these 37% participants would choose another profession if given the opportunity. In another study conducted in LMDC, it was found that 51.3% students were internally motivated however they also reported limited options for post-graduation.^16^ Out of the total participants, 32.5% participants did not find the dental profession worth leaving the country and facing the language and cultural barriers for it.

Results of our study can be compared with other local and international studies. In a similar study conducted on medical students in Pakistan, it was found that 46% of students had strong motivation whereas only 4% had low motivation.^7^ Similarly high levels of motivation were found in medical students in India.^17^ However contrary results were seen in a Chinese longitudinal study in which it was found that all types of motivation level of students declined over time.^10^ Similar results were reported in another Dutch study in which motivation levels of the students decreased by the time they reached final year although they were initially motivated to attend medical school.^18^

Several factors may have affected our results. Literature shows that the results were significantly different when a comparison was done between private and public medical schools.^7^ This can be attributed to the criteria for admission in both sectors which may vary. However no significant difference in motivation levels between private and public medical colleges of Khyber Pakhtunkhwa was found.^9^ Researchers believe that the strength of motivation questionnaire should be used more as a tool by faculty members and policy makers to help students who may need to be motivated.^19^ It was also reported in a French study that the SMMS questionnaire is a suitable tool to be used as a guide to strengthen motivation in students.^20^

### Limitations

A major limitation of our study was that data was collected from a single institute and comparison of motivation with other dental colleges is missing. A comparison of different institutes will give a better picture.

## CONCLUSION

Motivation plays an important role in achieving excellence in the professional studies. The role that motivation plays in pursuing success in professional studies, especially in the rigorous discipline of dentistry, is noteworthy. It is important to recognize that measuring motivation and success is a complex process, and that no one criterion can fully represent the complexity of these concepts. There should be a holistic strategy that takes into account many aspects of success rather than depending only on scoring criteria. We must strengthen our dedication to helping dentistry students fulfil their academic goals.

### Recommendation

It is recommended by the authors that such studies should be conducted at the start of the academic year when students are most motivated. A series of studies should be conducted which should focus more on outcome of motivation in terms of success and academic performance.

### Authors’ Contribution:

**FA:** Literature search, Data collection, Data analysis, Write-up, Proofreading and the accuracy of the study.

**MTE:** Conceptualization of study, Literature search, Write-up.

**FS:** Literature search, Data analysis, Data interpretation, Write-up.

**MF:** Literature search, Write-up.
